# A unique neural and behavioral profile of social anxiety in autism

**DOI:** 10.21203/rs.3.rs-6514342/v1

**Published:** 2025-05-28

**Authors:** Yu Hao, Sarah Banker, Matthew Schafer, Ember Zhang, Sarah Barkley, Jadyn Trayvick, Arabella Peters, Abigaël Thinakaran, Christopher McLaughlin, Xiaosi Gu, Jennifer Foss-Feig, Daniela Schiller

**Affiliations:** Icahn School of Medicine at Mount Sinai; NYU Grossman School of Medicine; Columbia University; University of Pennsylvania; Icahn School of Medicine at Mount Sinai; Icahn School of Medicine at Mount Sinai; Montclair State University; Icahn School of Medicine at Mount Sinai; Columbia University Irving Medical Center; Icahn School of Medicine at Mount Sinai; Icahn School of Medicine at Mount Sinai; Icahn School of Medicine, Mount Sinai

**Keywords:** autism spectrum disorder, social anxiety, social cognition, power, amygdala volume

## Abstract

Social anxiety (SA) commonly co-occurs with autism spectrum disorder (ASD), each sometimes misdiagnosed as the other. We examine behavioral and neural profile of SA in ASD in an online study (ASD = 575, control = 357) and a neuroimaging study (ASD = 72, control = 72). Using a naturalistic social interaction task, we identified acquiescent behaviors in individuals with both SA and ASD compared to those with ASD or SA alone. The amygdala—previously linked to anxiety and ASD—was uniquely enlarged only in adults with both SA and ASD. Furthermore, larger amygdala volume was associated with acquiescent behaviors in ASD, a relationship that was enhanced when accounting for SA. These findings suggest that autistic adults with larger amygdala are more likely to experience SA and difficulties in power dynamics (dominance or control), highlighting unique phenotype of SA in ASD.

## Introduction

Autism spectrum disorder (ASD) is a lifelong neurodevelopmental condition that affects approximately 1–2% of the population^1,2^. Anxiety was noted as a feature in Kanner’s 1943 description of autism, alongside core symptoms such as social communication and interaction difficulties and restricted, repetitive behaviors^3^. Research estimates that 30% to 50% of individuals with ASD (mainly cognitively unimpaired) experience social anxiety^4–6^—four times the rate in the general population^7^. Despite its prevalence and considerate phenotypic overlap^8^, the causes of social anxiety in ASD remain poorly understood, and few studies have examined social anxiety in autistic adults. Anxiety symptoms may limit the benefits of social skills interventions^9,10^, which may increase challenges of employment and social life. Understanding social anxiety in ASD is crucial for improving diagnosis, developing effective treatments, and enhancing support for this population.

Is autism with social anxiety merely a combination of autism and social anxiety, or does it represent a distinct condition? The hallmark symptoms of social anxiety disorder include a fear of negative evaluation or judgment by others and avoidance of social situations that trigger anxiety^11^. Previous studies found that fear of negative evaluation is related to social anxiety in ASD to the same degree as in non-autistic individuals^4,12,13^. In individuals with both ASD and social anxiety, social avoidance may stem from diminished social motivation^14^, as highlighted in a meta-analysis^5^. However, while some individuals with ASD show little interest in social interaction and appear indifferent to others’ opinions, many are aware of their social difficulties, which can lead to social anxiety^8^. These individuals value peer approval as much as their typically developing peers and strongly desire social connections^15–17^. However, difficulties in understanding social cues or knowing how to respond can make social interactions challenging, leading to heightened fears of negative evaluation and increased social avoidance (see systematic review^18^). Thus, social anxiety in autism may arise from core autism symptoms and exhibit distinct behavioral characteristics.

To explore this, we employed a dynamic, role-playing task allowing for a naturalistic assessment of implicit social behavior^19^. Our task incorporated affiliation and power behaviors^20–22^, which define a “social space”—a cognitive map that supports flexible social behavior across abstract domains^23^. We first examine whether task-derived affiliation and power behaviors show unique differences in autism with social anxiety. Previous studies showed that non-autistic individuals with social anxiety tended to perceive themselves as having low social rank or behaving submissively^24–26^, but none has examined behavior manifestations in autism population. Autistic individuals are often more vulnerable to exploitation and may comply with unreasonable requests due to difficulty recognizing manipulation or fear of consequences if they refuse^27^. Studies estimate that 49% to 80% of autistic adults experience interpersonal victimization^28,29^. Those with very low social motivation may reduce affiliative behaviors, such as avoiding physical contact, limiting self-disclosure, and resisting social engagement. Some may compensate by displaying higher power behaviors, such as rejecting requests or refusing to help. In contrast, neurotypical (NT) individuals with social anxiety generally do not have social skill deficits (for example they engage in greater perspective-taking and empathic functioning), yet tend to underestimate their own competence^30^. If social anxiety presents differently in individuals with and without ASD, we expect distinct social tendencies (i.e., affiliation and/or power behaviors) in each group.

Early studies on amygdala lesions suggested that the amygdala is a key component of the neural circuitry underlying social behavior, a theory formalized by Brothers in 1990^31^. Later, Baron-Cohen and colleagues (2000) proposed the “amygdala theory of autism,” suggesting that structural and functional abnormalities in this brain region may contribute to the social impairment characteristic of autism^32^. However, Amaral et al. (2003) argued that if the amygdala is dysfunctional in autism, it may be more strongly associated with heightened fear and anxiety rather than social deficits^33^. Most research on the relationship between amygdala volume and anxiety in autism has focused on children and adolescents. Some studies suggested that autistic individuals with anxiety had larger amygdala volumes than autistic individuals without anxiety or NT controls^34–36^, while a few reported smaller volumes^37^ or no significant differences^38^. Additionally, enlarged amygdala volumes have been observed in NT individuals with self-reported or clinically diagnosed anxiety^39^. Despite these findings, few studies have specifically examined the role of amygdala volume in the relationship between autism and social anxiety (see^40^). To our knowledge, no research has explored this connection in autistic adults—a gap this paper seeks to address.

**Figure 1:**
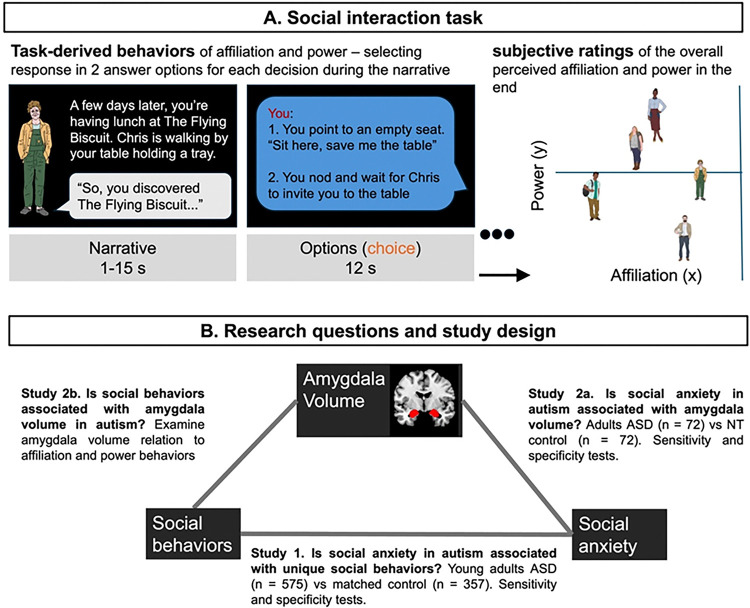
Overview of research questions and study design. (A) Participants engaged in a dynamic, role-playing game simulating social scenarios: in Study 1, they started a new school, navigated their way around, joined a club, and settled in, and in Study 2, they explored a new town to secure employment and housing. Both tasks were designed to parallel each other in the number and types of interactions and the roles of the characters. Illustrated characters with distinct social roles (e.g., an old acquaintance or potential employer) appeared on slides. Participants responded using button presses, selecting higher (+1) or lower (−1) affiliation or power behaviors. The power trials and affiliation trails in the task represent different scenarios of dominance/control or physical contact/social engagement. For example, in the power trial shown in the figure, response 1 to “Chris” is recorded as +1, while response 2 is recorded as −1. Some other power trials are: You wait for Chris to take the middle seat (+1) or you immediately move over to the middle seat (−1). Maya says to you: “there is a phone number on their website.” You go to the website on your phone (−1) or you say, “give them a call and schedule a meeting, please (+1).” Some example affiliation trials are: You say “So, what’s the deal with Maya? Catch me up! (+1) or you continue looking at your phone (−1). Chris goes in for a hug. You shake his hand instead (−1) or you hug him for a long moment (+1). Across the task, participants completed 30 affiliation trials and 30 power trials (6 trials per character). In the analysis, for each participant, we averaged all trails across characters in affiliation and power trials respectively. After the task, participants rated their perceived relationships with each character on a 2-D space based on affiliation (friendship or intimacy) and power (dominance or control). (B) Study 1 recruited online sample of ASD (with social anxiety n = 145, without social anxiety n = 430), examining how task-derived power and affiliation behaviors predicted self-reported social anxiety diagnoses (yes or no). Sensitivity analyses tested the robustness of findings by adding covariates (e.g., perceived social relations), removing participants with cognitive delays, reanalyzing data using different methods such as bootstrapping, etc. We also conducted specificity tests to confirm that these social behavior changes were specific to social anxiety rather than other frequently co-occurring conditions, such as depression or generalized anxiety. Results were compared with a neurotypical control-matched sample, matching by sex, age and education to the subset of ASD individuals who did not have any cognitive delays. Study 2 recruited autistic participants for both neuroimaging scans and behavioral data collection (with social anxiety n = 15, without social anxiety n = 57), following similar procedures to examine the relationship between amygdala volume and self-reported social anxiety diagnoses. Finally, we examined the relationship between social behaviors and amygdala volume.

It may be also the case that amygdala volume is associated with social behaviors in autism. The amygdala may encode social behaviors that are salient to social anxiety in autism. Previous research links amygdala reactivity to social cues with anxiety symptoms in autism^41,42^. While amygdala volume has been associated with individual differences of social network size^43^ and tolerance of social inequality^44^, its role in our task-derived affiliation-power behaviors remains unclear. Here, we analyzed self-reported social anxiety diagnoses as a vulnerability index in two studies, one utilizing a large online sample (ASD = 575, NT = 357) and a second with onsite neuroimaging (ASD = 72, NT = 72), both capturing social behaviors in a social interaction task. All comparisons were made on the basis of measurements taken from distinct samples. Figure 1 provides an overview of our research questions and study design.

## Results

### Study 1: Is social anxiety in autism associated with unique social behaviors?

In study 1, we analyzed data from 575 young adults (71% females, M_age_ = 25.38, S.D. _age_ = 3.31) who disclosed professional autism diagnoses. Of these, 145 (25.22%) also reported social anxiety (SA) disorder diagnoses. Demographic information and statistics of main research variables are provided in Supplementary Table S1. Individuals’ attestations of having SA diagnoses were further supported by significantly higher rates of self-reported social avoidance behavior (Supplementary Fig. S1).

Consistent with our hypothesis that autism with SA would show distinct social behaviors compared to autism without SA, our analysis found that a higher likelihood of self-reported SA was significantly associated with acquiescence (lower task-derived power behaviors, such as following commands or not in control of social situation) (χ^2^ = 4.30, *β* = −0.21, S.E. = 0.10, 95% CI = [−0.42, −0.01], p = 0.038). Affiliation behaviors were not related to SA (χ^2^ = 0.46, *β* = −0.07, S.E. = 0.10, 95% CI = [−0.26, 0.13], p = 0.455). Further validation analyses were conducted for sensitivity (i.e., testing with covariates of perceived social relations with game characters that was measured right after the task, and testing in a subset of those without self-reported cognitive delays), robustness (i.e., reverse the independent variable and the dependent variable, and bootstrapping methods; see Supplementary Material 1.3) and specificity (i.e., testing task-derived social behaviors relation to major depressive disorder (MDD) and generalized anxiety disorder (GAD)) of the results. Table 1 presents the statistics of the original analysis model along with sensitivity and specificity tests. These results consistently show that autistic individuals with SA exhibited more acquiescent behaviors than those without SA, and acquiescent behaviors were specifically related to SA but not any other co-occurring and overlapping conditions such as MDD or GAD.

**Figure 2:**
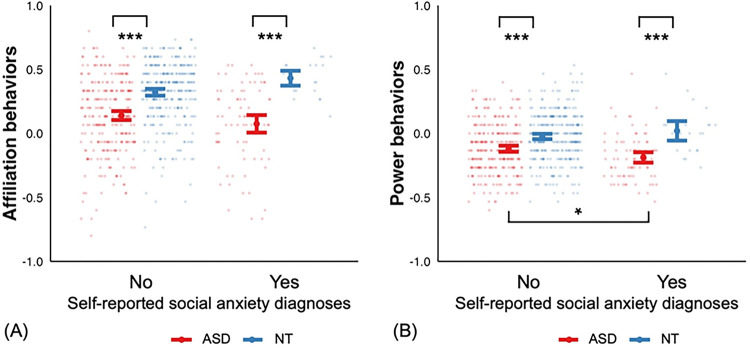
Relationship between social behaviors and self-reported SA diagnoses in ASD and NT groups. Sample size and mean in each group: ASD with SA n = 79, affiliation mean = 0.08, power mean = −0.19; ASD without SA n = 278, affiliation mean = 0.14, power mean = −0.12; NT with SA n = 25, affiliation mean = 0.43, power mean = 0.02; NT without SA n = 332, affiliation mean = 0.32, power mean = −0.02. (A) Affiliation Behavior Model: A regression model was used to predict task-derived affiliation behaviors, including an interaction term for group (ASD vs. NT) and SA (yes vs. no), with sex, age and education as covariates. The ASD group showed significantly lower task-derived affiliation behaviors than the NT group (F = 96.10, β = 0.19, S.E. = 0.02, p < 0.0001). There was also a significant interaction between group and SA (p = 0.028). (B) Power Behavior Model: A regression model was used to predict task-derived power behaviors, following the same structure as the affiliation behavior model. There was a significant main effect of group, indicating that the ASD group exhibited lower task-derived power behaviors than the NT group (F = 50.09, β = −0.10, S.E. = 0.02, p < 0.0001). Additionally, a significant interaction between group and SA was observed (p = 0.028). Note: task-derived affiliation behavior scores greater than 0 indicate a preference for affiliative choices during the task; task-derived power behavior scores of 0 indicates balanced decision-making, where participants equally distributed power between themselves and game characters. Negative values reflect a tendency to give power to others more frequently. Error bars represent 95% confidence intervals. *** p < 0.001, ** p < 0.01, * p < 0.05.

How do social behaviors in individuals with SA differ between individuals with and without ASD? To explore this, we compared our findings with a matched neurotypical (NT) control group (see Supplementary Table S2 for demographic comparisons). Overall, the ASD group showed significantly lower task-derived affiliation and power behaviors compared to NT (Fig. 2). Additionally, the interaction between group and SA was significant for both affiliation (p = 0.028) and power behaviors (p = 0.028). This suggests that the impact of SA on social behaviors differed between ASD and NT. Post-hoc analyses revealed that ASD individuals with SA had the most acquiescent behaviors among all groups (ASD without SA, NT with and without SA). By contrast, in NT group, SA did not significantly relate to both dimensions of social behaviors. There was no evidence for influence of specific game characters, as indicated by a non-significant three-way interaction between group, SA, and game characters (p > 0.33)

Unlike task-derived social behavior, perceived social relations with game characters that was measured after the task (see Fig. 1) did not significantly differ in individuals with SA compare with those without SA in both groups (Fig. S2). Together, these findings suggest that while observed social behaviors differed between SA in ASD and NT, their perception of social relationships remained similar.

### Study 2: Are social anxiety and social behavior in autism associated with amygdala volume?

#### Social anxiety

In study 2, we analyzed data from 72 individuals who completed both the behavioral task and 7T MRI, and had clinically confirmed ASD (52.8% females, M_age_ = 26.85, S.D. _age_ = 7.74, IQ = 67 to 140). Of these, 15 (21%) also disclosed SA diagnoses. Demographic information and statistics of main research variables are provided in Supplementary Table S3. Individuals’ attestation of having SA diagnoses in Study 2 was also further supported by significantly higher rates of self-reported social avoidance behavior (Supplementary Fig. S3).

**Figure 3:**
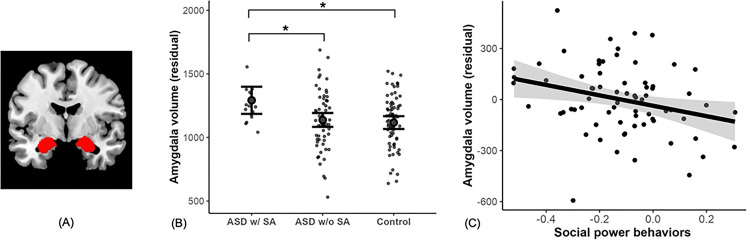
Amygdala volume relation to social anxiety and social power behaviors in autism. (A) Amygdala atlas. (B) Relation between amygdala volume (averaged left and right) and self-reported SA diagnoses in ASD and NT groups. Sample size and mean in each group: ASD with SA n = 15, amygdala volume mean = 1253; ASD without SA n = 57, amygdala volume mean = 1133; NT control n = 72, amygdala volume mean = 1130. A regression model was conducted predicting amygdala volume by Group (ASD w/SA, ASD w/o SA, and NT) and other covariates including sex, age and education. Post-hoc analysis showed that larger amygdala in ASD w/SA than ASD w/o SA (t = 2.60, p = 0.028) and NT (t = 2.86, p = 0.014). Amygdala volume didn’t differ in ASD w/o SA from NT (t = 0.53, p = 0.857). (C) Relation between amygdala volume and task-derived power behaviors in autism sample. A regression model was conducted predicting amygdala volume by task-derived power and affiliation behaviors, and other covariates including sex, age and education. It shows that larger amygdala volume corresponds to acquiescent behaviors. Note: Error bars and shaded area represent 95% confidence intervals. *** p < 0.001, ** p < 0.01, * p < 0.05.

Consistent with our hypothesis that amygdala volume would be linked to social anxiety in autism, we found that self-reported SA was significantly associated with larger amygdala volume (χ^2^ = 5.55, *β* = 0.88, S.E. = 0.41, 95% CI = [0.07, 1.69], p = 0.018; Fig. 3). Further validation analyses were conducted for sensitivity (i.e., testing with covariates of intracranial volume (ICV) and anterior cingulate cortex volume (ACC) as a control region (see Methods)), robustness (i.e., reverse the independent variable and the dependent variable, and bootstrapping methods, see Supplementary Material 2.3) and specificity (i.e., testing amygdala volume relation to MDD and GAD) of the results. Table 2 presents the statistics of the original analysis model along with sensitivity and specificity tests. These results consistently show that autistic individuals with SA had larger amygdala volume than those without SA, and larger amygdala volume were specifically related to SA but not any other co-occurring and overlapping conditions such as MDD or GAD.

We next asked whether amygdala volume in autistic individuals with and without SA differs from NT individuals. Since Study 1 found no significant differences in task-derived social behaviors between NT individuals with and without SA in the online population, we combined the entire in-person NT group for comparison with the ASD group for the analysis of amygdala volume to enhance statistical power. We compared amygdala volume across the 3 groups: ASD with SA (n = 15), ASD without SA (n = 57), and a NT group (n = 72, 62.5% females, M_age_ = 25.89, S.D. _age_ = 6.44, IQ = 76 to 135; within this group income, or the proportion of individuals with SA diagnoses (see Table S4 for demographic comparisons). Results showed that individuals with co-occurring ASD and SA had significantly larger amygdala volumes compared to those with ASD without SA and the NT group (p = 0.016, Fig. 3). Further analysis showed that amygdala volume differentiated SA only within the ASD group, but not in NT individuals (p = 0.242), likely due to limited statistical power.

#### Social behavior

Thus far, we have shown that self-reported SA was uniquely linked to acquiescent behaviors (Study 1) and larger amygdala volume (Study 2) in ASD. We therefore asked whether amygdala volume is associated with social behaviors in ASD.

Consistent with this prediction, we found that larger amygdala volume was associated with lower task-derived power behaviors (F = 6.47, *β* = −0.35, S.E. = 0.14, 95% CI = [−0.63, −0.08], p = 0.013), but not task-derived affiliation behaviors (F = 3.13, *β* = −0.23, S.E. = 0.13, 95% CI = [−0.49, 0.03], p = 0.081). These results remained significant after controlling for ICV, ACC and psychiatric medication use. Furthermore, when self-reported SA was added as a covariate, the association between power behaviors and amygdala volume strengthened by 15% (F = 9.38, *β* = −0.41, S.E. = 0.13, 95% CI = [−0.67, −0.14], p = 0.003) but the association between affiliation behaviors and amygdala volume only changed for 3% (F = 3.68, *β* = −0.24, S.E. = 0.12, 95% CI = [−0.48, 0.01], p = 0.060). These results suggested that acquiescent behaviors and social anxiety uniquely contribute to variations in amygdala volume, indicating distinct underlying mechanisms for each.

## Discussion

Social anxiety in autism has been hypothesized to be closely tied to core ASD symptoms^5,45–47^. However, its unique behavioral or biological correlates remain unclear. Our findings show that autistic individuals with co-occurring SA exhibited significantly lower task-derived power behaviors compared to both autistic individuals without SA and NT individuals, regardless of SA. Notably, this pattern was only observed during the task and did not align with their self-perceptions of social interaction during the task. Furthermore, although SA also highly comorbid with MDD and GAD in autism, lower task-derived power behaviors were not associated with co-occurring MDD or GAD, suggesting that autism with SA presents a distinct behavioral profile of social acquiescence. At the neural level, autistic individuals with SA exhibited enlarged amygdalae compared to both those without SA and the overall NT control group. This enlargement was specific to SA, as it was not observed in autism with MDD or GAD. Moreover, larger amygdala volume was linked to acquiescent behaviors in ASD, both in absolute size and relative to whole-brain volume. Overall, these findings highlight distinct behavioral and neural features of autism with SA, emphasizing the need for more precise diagnostics and tailored interventions for this subgroup.

Autistic individuals often engage in acquiescent behaviors, feeling pressured to comply with others’ requests—some to avoid causing trouble, some out of naivete, others out of fear of consequence^27^. This may stem from difficulties in processing power dynamics, as autistic individuals judge power relationships more slowly than NT peers, suggesting a more deliberate, explicit processing style^48^. Consequently, these might be the reasons why autistic adults were perceived as warmer but less competent than neurotypical individuals^49^. Our findings indicate that SA not only amplifies acquiescent behaviors in ASD, but also further widens the behavioral differences between ASD and NT who reported SA (Figure 2A). Our study found no significant differences in affiliation or power behaviors between NT with and without SA. In NT individuals, SA may be primarily associated with cognitive symptoms such as a fear of negative evaluation, whereas in ASD, SA may be related to a lack of social skills or reduced interest in social engagement^5,8,18^. As a result, White et al. (2014) argued that SA in ASD is often inferred from observable behaviors rather than explicitly expressed thoughts as with NT individuals^8^.

While acquiescence may serve as a coping mechanism for forming relationships, over-reliance on it may lead to distress, increased workload, or even exploitation and betrayal. As explained by the dialectical misattunement hypothesis, autism may not be a disordered function of individual brains but a mismatch of interpersonal dynamics^50^. Over time, these accumulated negative experiences may contribute to the development or worsening of social anxiety in autism.

In the brain, we discovered that amygdala size plays a key role in autism with SA. The amygdala is related to safety detection^51^, but individuals with autism may lack of effective safety detection in social contexts^52^. Research until now has primarily focused on autistic children, yielding mixed results but primarily reporting larger amygdalae related to anxiety^34–36^. Due to differing developmental changes in amygdala size in ASD and NT controls^35,53^, it is important to examine the role of the amygdala in social anxiety among autistic adults. Our findings contribute to filling this gap, showing that larger amygdala volume is linked to self-reported SA in autism. A recent meta-analysis found increased right amygdala volume in autism compared with non-autistic children and adults with no cognitive disabilities^54^. Our findings suggest that enlarged amygdalae in autistic adults compared to NT individuals primarily occur in those with SA, indicating that prior reports of increased amygdala volume in ASD may be driven by this specific subgroup (c.f.^40^). This indicates that co-occurring SA predicts a heterogeneous pattern of neuropathology in autism.

We also show that larger amygdala volume was associated with acquiescent behaviors in autism, supporting the “amygdala theory of autism,” which suggests that differences in amygdala volume may underlie autism-related social impairments^32^. When accounting for SA as a covariate, the association between amygdala and SA was further enhanced. These results suggest that despite the link between acquiescent behaviors and social anxiety (study 1), each of these factors seem to uniquely related to variations in amygdala volume, indicating distinct underlying mechanisms for each. Prior evidence suggests that amygdala hypoactivation in ASD may be masked by co-occurring anxiety, further complicating its role in social processing^55^. Given the amygdala’s role in integrating emotional and social information, its function cannot be fully understood within either domain alone^55^. In our study, the statistically enhanced association between acquiescent behaviors and amygdala volume when controlling for SA aligns with previous findings on amygdala function, suggesting that social processing, social anxiety, and ASD involve both shared and distinct neural pathways.

Both study 1 and 2 showed that the ASD group exhibited significantly lower task-derived affiliation behaviors than the NT group. These results are consistent with our previous report using a separate online sample as well as a partially overlapping onsite sample^56^ (see Methods). The current study offers novel insights by differentiating task-derived affiliation behaviors in ASD versus NT, and showing that social anxiety may further amplify these group differences (Figure 2B), particularly in individuals without cognitive delays. However, we also found that affiliation behaviors were not associated with amygdala volume in ASD, despite prior findings from a partially overlapping onsite neuroimaging sample showing a link between amygdala volume and clinician-rated ASD symptoms^57^. This suggests that affiliation behaviors may not be as directly tied to the neural substrates of both social anxiety and core ASD symptoms as acquiescent behaviors are.

In Study 2, we did not detect the effect observed in Study 1 of lower task-derived power behaviors in ASD compared to the NT group (trending, p = 0.10). Based on the effect size from Study 1 (Cohen’s d = −0.36), a total sample size of at least 248 would be needed to achieve 80% power (α = 0.05) to detect lower task-derived power behaviors in individuals with both SA and ASD compared with those ASD without SA. This calculation assumes that SA comprises 22% of the ASD sample (excluding individuals with cognitive delays). Although the effect size for acquiescent behaviors related to social anxiety is small, acquiescent behaviors were nevertheless positively associated with amygdala volume with a medium effect size (Cohen’s f^2^ = 0.19), suggesting that this behavioral effect may hint at broader underlying neurobiological differences.

Our study has several limitations. First, its cross-sectional design prevents us from determining causal relationships between social anxiety, power behaviors, and amygdala volume. Longitudinal research is needed to explore whether early-life social behaviors contribute to social anxiety or if social anxiety leads to reduced power behaviors. Tracking amygdala volume changes over time could also clarify whether power behaviors influence amygdala structure or vice versa. Second, we relied on self-reported social anxiety diagnoses and ASD diagnoses in Study 1 rather than clinician assessments, which may introduce bias. However, self-reported SA was correlated with social avoidance and clinician-endorsed autism severity in our study, supporting its relevance. Future research should incorporate clinician-verified SA and ASD diagnoses to strengthen validity. Third, our findings may not fully generalize to the broader ASD population. We excluded individuals with intellectual disability when comparing with NT in study 1, where social anxiety is most prevalent^58^. Our onsite ASD sample had higher education and IQ, while the online sample differed in social avoidance, autism traits, and education levels, with more females. Despite these differences, both samples replicated known associations between social avoidance and social anxiety, and we found no sex differences in key results. Future studies should include more diverse ASD samples with a broader range of social avoidance, education levels, and IQ to improve generalizability.

## Conclusion

Social challenges in autism often intensify in adulthood, leading to negative outcomes, particularly for those with co-occurring ASD and social anxiety^4^. Understanding the causes and correlates of social anxiety in autism is essential for developing targeted interventions. Our findings highlight unique behavioral and neural features of social anxiety in autism—acquiescent behavior and larger amygdala volume. Notably, amygdala volume emerged as a neural marker related to both acquiescence and social anxiety, suggesting a potential shared etiology. Future research should examine biological and environmental factors influencing acquiescent behaviors to better understand the heterogeneity of autism with social anxiety. Integrating structural/general neuroimaging with naturalistic social paradigms could refine diagnostics and distinguish social anxiety in autism from that in neurotypical populations. Practically, these insights could inform targeted interventions, particularly social power behavior training, to enhance social competence and strengthen social connections for autistic individuals with social anxiety.

## Methods

### Participants

#### Study 1 - Online behavior sample

We recruited a large ASD participants sample through Simons Powering Autism Research (SPARK) Research Match program, a project supported by Simons Foundation. All registered SPARK participants self-attested to having received professional diagnoses of ASD. The eligibility criteria were: (1) ages 18 to 30, (2) Social Communication Questionnaire^59^ total score > 9. Among 956 participants who consented, 675 individuals completed all surveys and the social task. The sample size after excluding 100 participants with an “ASD validity flag” (criteria include diagnosis rescinded, or diagnosis age earlier than 15 months, repetitive behavior scale-revised score < 10, unknown diagnosis source, etc.) identified in the SPARK phenotype dataset was 575 (409 females). IQ was measured by cognitive tests from TestMyBrain (https://www.testmybrain.org). All participants provided written informed consent and received compensation for their participation.

Neurotypical (NT) controls were recruited from Prolific (www.prolific.com), a platform that helps researchers recruit participants for their online research. The exclusion criteria were: (1) neurodevelopmental diagnoses, including ASD, dyslexia, attention deficit disorder or attention deficit hyperactivity disorder, (2) mild cognitive impairment or dementia, (3) history of neurological concerns such as epilepsy, traumatic brain injury, Parkinson’s disease, epilepsy, seizures, or multiple sclerosis, (4) other chronic diseases such as diabetes, heart disease or stroke, and (4) first language is not English. IQ was measured by ICAR (The international cognitive ability resource https://icar-project.com).

Autistic individuals without intellectual impairment were shown to experience more social anxiety^8^. Among the final ASD sample of 575, 200 had self-identified cognitive or language delays (such as intellectual disability, specific language impairment). Our sample showed similar rate of cognitive delays among ASD individuals as reported in the latest systematic review^2^. We matched NT samples to ASD samples removing those who self-reported cognitive or language delays. From a total sample size of 696 NT participants, we selected 357 participants who matched our ASD sample based on sex, age, and education using the “MatchIt” package in R. Among these 357 NT individuals, 25 (7.08%) declared SA diagnoses, consistent with the general US population^7^.

#### Study 2 - Onsite imaging sample

ASD participants were recruited through physical flyers around New York City, email listserv announcements, local research registry and word of mouth. The eligibility criteria were: (1) ages 18 to 50, (2) meeting DSM-5 criteria for ASD, (3) having an IQ over 60 (assessed onsite by the Wechsler Abbreviated Scale of Intelligence and Wechsler Intelligence Scale for Adults^60,61^, (4) having no history of neurological concerns like epilepsy or traumatic brain injury, and (5) no substance or alcohol abuse disorders no recreational drug use. ASD screening was conducted by licensed clinicians using the Autism Diagnostic Observation Schedule, 2nd edition (ADOS-2^62^), supplemented with developmental and clinical history as needed, to inform DSM-5 criteria. We intentionally over-recruited females to ensure better representation of this underrepresented group in autism research.

NT participants were recruited through announcements posted on physical flyers around New York City and email listservs with the eligibility criteria of (1) age between 18 and 50, (2) IQ >60 (assessed via The Wechsler Abbreviated Scale of Intelligence; WASI-II), (3) no 1st degree relatives with idiopathic ASD, (4) no history of neurological concerns (e.g., epilepsy, TBI), and (5) no recreational drug use 24 hours prior to MRI visit.

One hundred and sixteen autistic individuals with onsite clinically confirmed ASD and 92 NT individuals participated in the study. Among these participants, 101 and 77 for ASD and NT respectively completed the social interaction task with valid data (if they completed at least 75% of the trials and demonstrated above-chance accuracy on the post-task memory test) and have completed demographic data. Twenty-two participants from ASD and 5 participants from NT group did not complete MRI scanning, and 7 participants’ data was removed in ASD group due to unusable structural scan. After applying these criteria, the final sample consisted of 72 sex-balanced (52.8% female) adults with ASD and 72 NT (62.5% female) for volumetric analysis. Our current sample included all data from the previous study that examined depression in ASD^57^, along with 7 additional participants whose data were collected afterward. All participants gave written informed consent and received compensation for their participation.

The proportions of self-reported SA did not differ between the Study 1 online and Study 2 onsite ASD samples (p = 0.360, Two-Proportion Z-Test). There was also no age and self-reported autism symptoms differences between the two samples. However, compared to the Study 1 online sample, the Study 2 onsite sample had higher education and income levels, a greater proportion of males, and lower self-reported social avoidance.

Functional and structural MRI data and behavioral data from the social interaction task for a subset of ASD (n=65) participants has been reported elsewhere^57^. Here, we confirm the behavioral finding of lower affiliation in ASD in a larger neuroimaging group (data collection continued after previous publications^56,57^). Amygdala volume has been previously analyzed in the partially overlapping sample^57^ but was examined in relation to other measures and research questions.

### Assessment of social anxiety, autism symptoms and social behaviors

#### Self-reported Social Anxiety Disorder Diagnosis

Social anxiety was measured by a screening question: “Have you ever been diagnosed with any of the following psychiatric disorders?” Social anxiety disorder was one of the options along with other diagnoses such as major depressive disorder and generalized anxiety disorder. The response to this question is either “yes” or “no.”

#### Self-reported Social Avoidance

Social avoidance was measured by *Liebowitz Social Anxiety Scale,* which assesses how social anxiety affects participants’ lives across various situations^63^. It asks, “How often do you avoid the following situations?” Examples include speaking up at a meeting, expressing disagreement or disapproval to someone you don’t know well, or going to a party. Participants rated their avoidance on a 4-point scale: never (0), occasionally (1), often (2), and usually (3). The self-report version of this scale compares well to the clinician-administered version^64^ and shows good reliability and validity^65,66^. It is also shown to be reliable used in autistic adults with social anxiety disorder^67^.

#### Self-Rated Autism Symptoms

Self-rated autism symptoms were measured by the Broad Autism Phenotype Questionnaire (BAPQ), a self-report questionnaire for adults. This questionnaire has succeeded in meeting both sensitivity and specificity requirements for detecting the broader autism phenotype^68^. Its robust psychometric properties^68,69^ and absence of ceiling effects^70^ in individuals with ASD indicate that it performs effectively in both clinical populations^71^ and the general population.

#### Social Interaction Task

In this study, participants engaged with naturalistic game designed to map out their individual social space, tracing their interactive pathways with various characters^19^. We have two version of the game for Study 1 and Study 2. In Study 1, participants started a new school and needed to find their way around and settle in. Their task was to join a club and find a locker at their new school. In Study 2, participants find themselves in a new town, with the objective of securing employment and housing by interacting with local residents. The task structure, in terms of number and types of interactions and roles of characters, were designed to parallel the original task exactly. During gameplay, participants encountered different social characters, with conversation occurring via text bubbles. The characters possessed distinct attributes suggestive of their social roles—such as an old acquaintance, an assistant, or a potential employer. Participants navigated the social scenarios by choosing from two dialogue options, using a button press to dictate their responses. Some of these responses represent *social affiliation behaviors* with one option representing higher affiliation behavior (= +1) and the other option representing lower affiliation behavior (= −1). On the other hand, some of these responses represent *social power behaviors* – either being authoritative (= +1) or submissive (= −1). An example is shown in Figure 1. This interactive format allows participants to experience a consistent narrative while their choices actively influence the direction of the story, akin to “choose-your-own-adventure” games. There are 5 characters in the game, such as boss or assistant, and for each character, there are 6 trials of affiliation choices and 6 trials of power choices. Three additional trials involved neutral interaction with a control character. For analysis, we calculated the mean of affiliation and power choices across all trials to represent *affiliation behaviors* and *power behaviors, respectively.* After completing the task, participants were asked to rate their feelings of affiliation and power with those characters on a 2-D space. They were instructed that affiliation represented the friendship or intimacy they felt with each character and that power represented the control and dominance they felt each character possessed over them. We then calculated the mean of the affiliation perceptions and power perception across the 5 characters to represent *perceived affiliation* and *perceived power*.

### Neuroimaging acquisition and processing

Structural MRI data was acquired for all participants on a 7 Tesla whole body scanner (Magnetom, Siemens Healthcare, Erlangen, Germany). A SC72CD gradient coil was used with a single coil transmit and a 32-channel head coil (Nova Medical, Wilmington, MA, USA). A T1-weighted MP2RAGE sequence was performed on each participant, with a 0.7 mm × 0.7 mm × 0.7 mm voxel resolution. Field of view (FOV) was 225 × 183, orientation of scan was coronal, repetition time (TR) was 6000 ms and echo time (TE) was 3.62 ms. A coronal-oblique T2-weighted turbo spin echo (T2-TSE) sequence was also obtained for all participants, with a 0.43 mm × 0.43 mm × 2.0 mm voxel resolution. FOV was 222 × 177, orientation of scan was coronal, TR was 9000 ms and TE was 69 ms.

Results included in this manuscript come from preprocessing performed using *fMRIPrep* 22.0.0 (RRID:SCR_016216)^72,73^, which is based on Nipype 1.8.3 (RRID:SCR_002502)^74,75^. One T1-weighted (T1w) image was found within the input BIDS dataset. The T1-weighted (T1w) image was corrected for intensity non-uniformity (INU) with N4BiasFieldCorrection^76^, distributed with ANTs 2.3.3 (RRID:SCR_004757)^77^, and used as T1w-reference throughout the workflow. The T1w-reference was then skull-stripped with a *Nipype* implementation of the antsBrainExtraction.sh workflow (from ANTs), using OASIS30ANTs as target template. Volume-based spatial normalization to two standard spaces (MNI152NLin2009cAsym, MNI152NLin6Asym) was performed through nonlinear registration with antsRegistration (ANTs 2.3.3), using brain-extracted versions of both T1w reference and the T1w template. The following templates were selected for spatial normalization: *ICBM 152 Nonlinear Asymmetrical template version 2009c*^81^[RRID:SCR_008796; TemplateFlow ID: MNI152NLin2009cAsym], *FSL’s MNI ICBM 152 non-linear 6th Generation Asymmetric Average Brain Stereotaxic Registration Model*^82^[RRID:SCR_002823; TemplateFlow ID: MNI152NLin6Asym]. FreeSurfer (http://surfer.nmr.mgh.harvard.edu) automated segmentation of the volumes was used to extract amygdala volume. We averaged the bilateral amygdala volumes as amygdala volume for the subsequent statistical analysis.

### Statistical Analyses

#### Study 1: Social Behaviors in Autism and Social Anxiety.

To examine whether autistic individuals with SA exhibit distinct social behaviors, we conducted a logistic regression predicting SA diagnosis (yes vs. no) using task-derived power and affiliation behaviors as independent variables. Covariates included sex, age, SES, self-reported cognitive delays, and IQ. For sensitivity analyses, we added perceived affiliation and power, as well as self-reported MDD and GAD as additional covariates. We repeated the analysis in a subset excluding individuals with cognitive delays. To assess the robustness of our findings, we applied bootstrapping. To test specificity, we substituted SA with MDD or GAD as the dependent variable while keeping independent variables constant.

To compare findings with the NT group, we matched NT participants to ASD participants without cognitive delays on sex, age, and education. We ran two regression models predicting task-derived power and affiliation behaviors, with interaction terms for Group (ASD vs. NT) and SA (yes vs. no), controlling for sex and age. P values of the interation were FDR-corrected for multiple comparisons. These models were repeated for perceived affiliation and power behaviors. Moreover, to explore if the results were driven by specific game character, we additionally added game character in the interaction model as a 3-way interaction.

#### Study 2: Amygdala Volume, Social Anxiety and Social Behaviors in Autism.

To assess whether autistic individuals with SA exhibit distinct amygdala volume differences, we conducted a logistic regression predicting SA (yes vs. no) using amygdala volume as independent variable. Covariates included sex, age, SES, and IQ. Sensitivity analyses controlled for ICV, ACC volume (since our previous research identified ACC differences in ASD with co-occurring depression^57^, we included the ACC as a control region for comparison), and self-reported MDD and GAD. Bootstrapping was applied for robustness testing. To test specificity, we substituted SA with MDD or GAD while keeping independent variables unchanged.

To compare ASD and NT groups, we used a NT sample that was not statistically different from ASD sample on sex, age, and income. A regression model predicted amygdala volume based on Group (ASD with SA, ASD without SA, and NT), controlling for sex, age, education, and ICV. As an exploratory analysis, we examined differences in NT individuals with and without SA by including an interaction term for Group (ASD vs. TD) and SA (yes vs. no). Furthermore, in ASD group, we tested whether amygdala volume was predicted by task-derived power and affiliation behaviors, controlling for sex, age, education, IQ.

All statistical tests used were two-sided and report test statistics, standardized beta coefficients (where applicable), and adjusted p-values. For interaction terms, estimated marginal means were computed using the “emmeans” package in R. Post-hoc pairwise comparisons were conducted using Tukey’s method to control for family-wise error.

## Figures and Tables

**Table 1.** Standardized beta coeffi cients (standard errors) of logistic regression models predicting self-reported diagnoses (two levels, YES vs. NO) by social behaviors and demographic covariates as well assensitivity and specifi city tests in the online autism sample (n = 575).

		Original model	Sensitivity tests			Specificity tests	
Dependent variable		SA YES n = 145 NO n = 430	SA YES n = 79 NO n = 278 remove those with delays	SA YES n = 145 NO n = 430	SA YES n = 145 NO n = 430	MDD YES n = 371 NO n = 204	GAD YES n = 445 NO n = 130
*Independent variables*	**Power behavior**	**−0.21 (0.10)** *	**−0.32 (0.14)** *	**−0.21 (0.11)** *	**−0.21 (0.11)** *	−0.13 (0.10)	0.03 (0.11)
	Affiliation behavior	−0.07 (0.10)	−0.07 (0.13)	−0.08 (0.10)	−0.06 (0.10)	0.01 (0.10)	−0.09 (0.11)
	Sex	**0.58 (0.24)** *	**0.78 (0.37)** *	**0.58 (0.24)** *	0.19 (0.25)	**1.21 (0.20)** ***	**1.42 (0.22)** ***
	Age	−0.02 (0.03)	0.02 (0.04)	−0.02 (0.03)	−0.03 (0.03)	0.05 (0.03)	0.02 (0.03)
	Education	**−0.12 (0.06)** *	**−0.20 (0.08)** *	**−0.12 (0.06)** *	−0.06 (0.06)	**−0.25 (0.06)** ***	**−0.20 (0.06)** **
	IQ	−0.03 (0.02)	−0.02 (0.03)	−0.03 (0.02)	**−0.04 (0.02)** *	0.03 (0.02)	0.01 (0.02)
	Cognitive delays	**−0.42 (0.21)** *		**−0.42 (0.21)** *	**−0.43 (0.21)** *	−0.01 (0.20)	−0.18 (0.23)
	Perceived power			0.07 (0.49)			
	Perceived affiliation			0.06 (0.31)			
	MDD				**0.57 (0.28)** *		
	GAD				**1.47 (0.43)** ***		

Note:

*** p < 0.001

** p < 0.01

* p < 0.05.

**Table 2.** Standardized beta coeffi cients (standard errors) of logistic regression models predicting self-reported diagnoses (two levels, YES vs. NO) by amygdala volume and demographic covariates as well assensitivity and specifi city tests in the onsite autism sample (n = 72).

		Original model	Sensitivity tests			Specificity tests	
Dependent variable		SA YES n = 15 NO n = 57	SA YES n = 15 NO n = 57	SA YES n = 15 NO n = 57	SA YES n = 15 NO n = 57	MDD YES n = 32 NO n = 40	GAD YES n = 34 NO n = 38
*Independent variables*	**Amygdala volume**	**0.88 (0.41)** *	**1.20 (0.49)** **	**1.22 (0.53)** **	**0.88 (0.44)** *	0.26 (0.30)	0.40 (0.31)
	Sex	0.55 (0.69)	−0.29 (0.88)	−0.30 (0.89)	−0.09 (0.81)	**1 53 10.60)** **	**1.56 (0.58)** **
	Age	−0.01 (0.05)	−0.03 (0.05)	−0.03 (0.05)	−0.04 (0.05)	0.08 (0.04)	−0.02 (0.04)
	Education	−0.42 (0.40)	−0.39 (0.43)	−0.38 (0.43)	−0.21 (0.44)	−0.56 (0.34)	−0.23 (0.33)
	IQ	−0.01 (0.02)	0.00 (0.02)	0.00 (0.02)	−0.02 (0.02)	0.03 (0.02)	**0.04 (0.02)** *
	ICV		−0.87 (0.57)	−0.87 (0.57)			
	ACC volume			−0.04 (0.38)			
	MDD				**1.92 (0.84)** *		
	GAD				0.30 (0.81)		

Note: Amygdala volume is the average volume of left and right amygdala. SES was calculated by the sum of normalized scores of education and income (ordinal variable).

*** p < 0.001

** p < 0.01

* p < 0.05.

## Data Availability

Data and code for this study is available at https://osf.io/2gzxw/?view_only=e0eda95675f84c5798e8460eb9eac01e
